# Supplementary material for: Metastability and Ostwald step rule in the crystallisation of diamond and graphite from molten carbon
Source: Nat Commun. 2025 Jul 9;16:6324. doi: 10.1038/s41467-025-61674-5 (PMC12241486; doi:10.1038/s41467-025-61674-5)
Supplement: Supplementary file 1 — Supplementary Information [file 41467_2025_61674_MOESM1_ESM.pdf]

Supplementary Information:
Metastability and Ostwald Step Rule in the Crystallisation of Diamond and Graphite from Molten Carbon

Davide Donadio,[1] Margaret L. Berrens,[1,2] Wanyu Zhao,[3] Shunda Chen,[3] and Tianshu Li[3]

[1)] *Department of Chemistry, University of California Davis*[a)]

[2)] *Quantum Simulations Group, Physics Division, Lawrence Livermore National Laboratory, Livermore, California 94550, United States.*

[3)] *Department of Civil and Environmental Engineering, George Washington University*

[a)] Electronic mail: ddonadio@ucdavis.edu.

SUPPLEMENTARY FIGURE 1

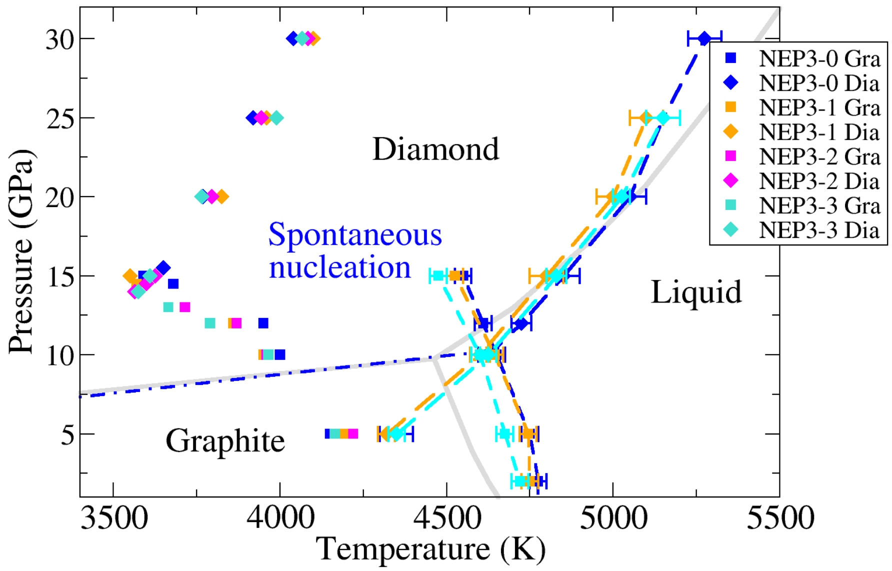

FIG. S1. Phase diagram and spontaneous crystallisation temperature with four different parameterisations of the neuroevolution machine learning potential (NEP type 3) trained on the LDA data set.

SUPPLEMENTARY FIGURE 2

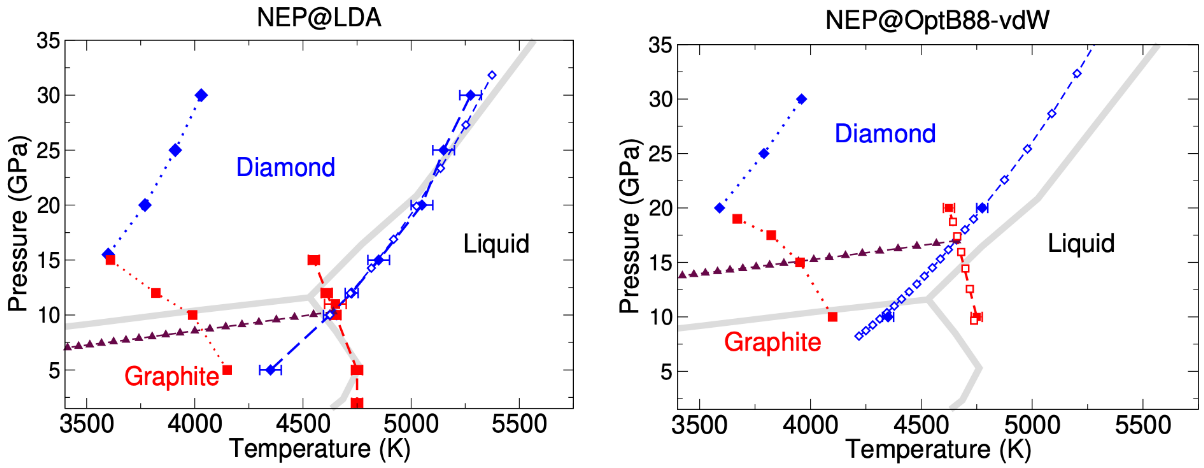

FIG. S2. Comparison between the phase diagrams and spontaneous crystallisation temperatures obtained using neuroevolution potentials trained on DFT datasets using the local density approximation[1] (LDA, left panel) and the OptB88-vdW functional[2,3] (right panel). Error bars for the graphite-liquid and diamond-liquid coexistence lines are computed as the difference between the lowest temperature at which the crystalline phase melts and the highest temperature at which it grows.

SUPPLEMENTARY FIGURE 3

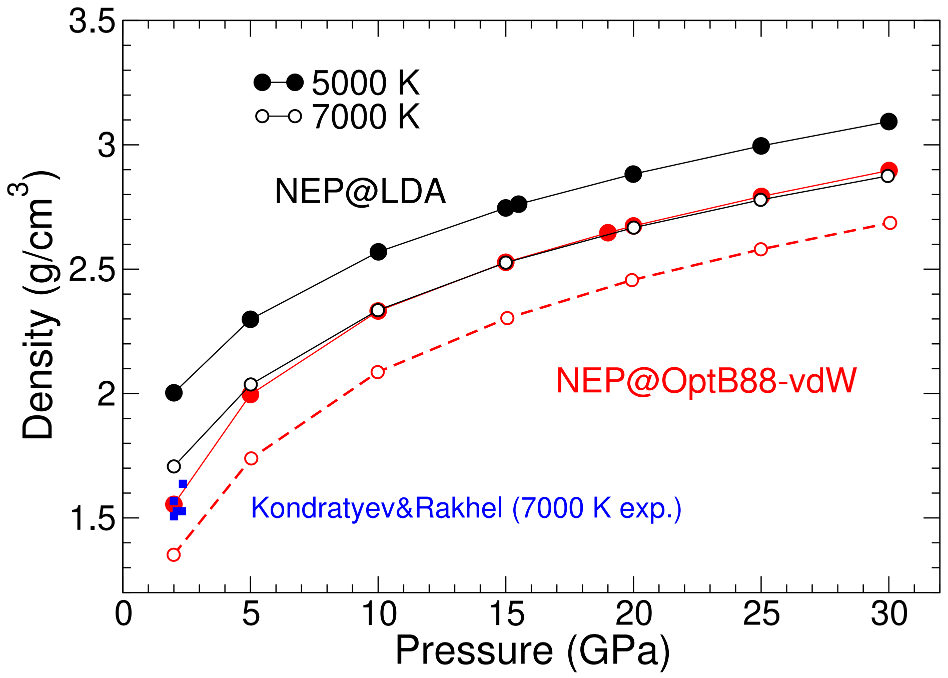

FIG. S3. Equation of state of liquid carbon at 5000 K (filled symbols) and 7000 K (empty symbols) computed with NEP@LDA and NEP@OptB88-vdW. Experimental measurements from Kondratyev and Rakhel[4] at 7000 K and up to 2 GPa are reported for reference. The differences between NEP@LDA and NEP@OptB88-vdW reflect the differences in the equation of state of liquid carbon computed by DFT using LDA and GGA functionals.[5] Data points at 15 and 15.5 GPa for NEP@LDA and at 19 and 20 GPa for NEP@OptB88-vdW show that there is no density discontinuity at the pressures at which the system transitions between crystallizing into graphite and diamond.

SUPPLEMENTARY FIGURE 4

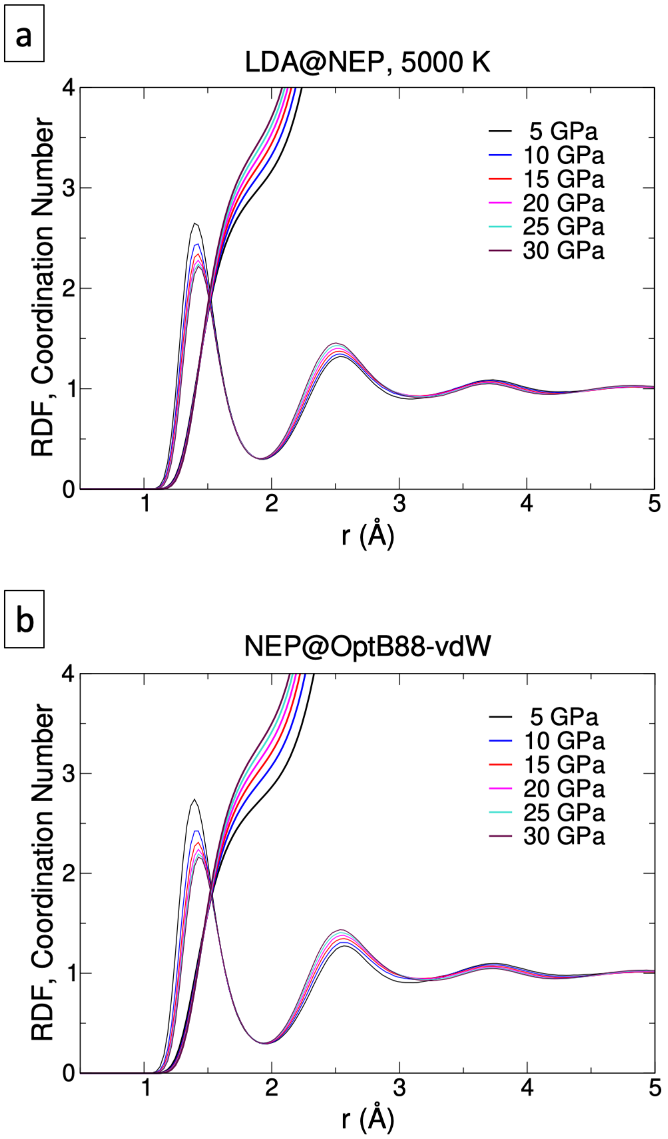

FIG. S4. Radial distribution functions and their volume integral of liquid carbon at 5000 K at different pressures modeled with NEP@LDA (a) and NEP@OptB88-vdW (b) potentials. The two models yield similar features, indicating gradual structural changes as a function of pressure. The intensity of the first peak indicates that NEP@OptB88-vdW liquid models are more structured than NEP@LDA models at the same pressure.

SUPPLEMENTARY FIGURE 5

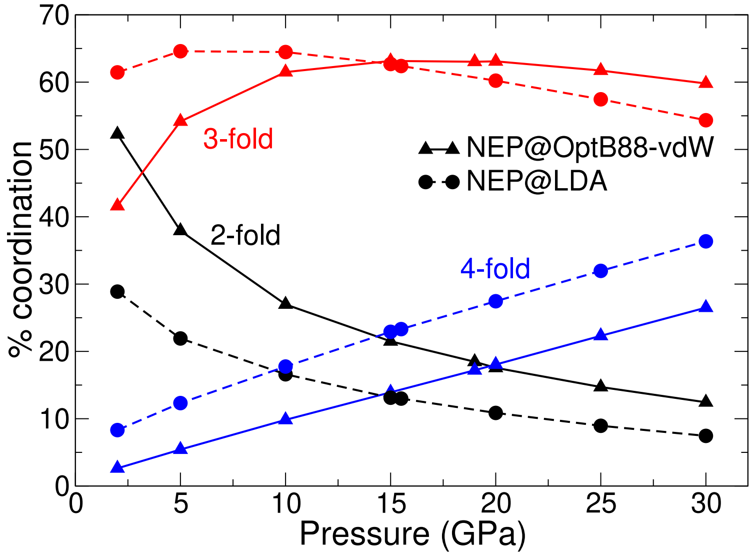

FIG. S5. Statistics of the local coordination (number of nearest neighbors within a 1.85 Å cutoff) in liquid carbon at 5000 K computed with NEP@LDA (circles) and NEP@OptB88-vdW (triangles). The number of 4-fold carbons increases linearly with pressure but does not exceed that of 3-fold carbons over the whole pressure range explored. The structural differences between the two liquids would be reduced to within statistical uncertainties by shifting the NEP@LDA data by a 6 GPa pressure offset. The data points at 15 and 15.5 GPa for NEP@LDA and 19 and 20 GPa for NEP@OptB88-vdW show that there is no structural discontinuity at the tipping pressure of diamond crystallisation.

SUPPLEMENTARY FIGURE 6

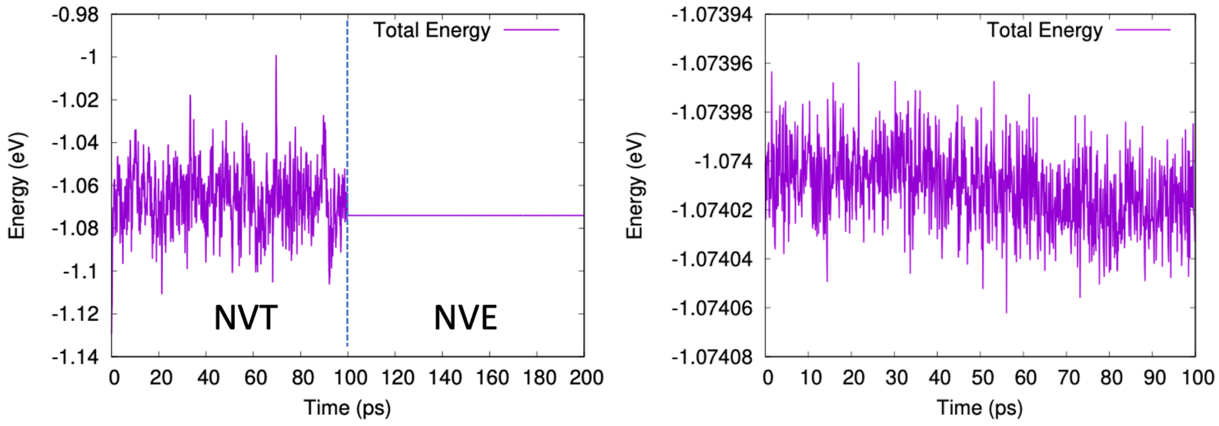

FIG. S6. Total energy in a MD simulation of liquid carbon at 4500 K. The left panel shows a 100 ps equilibration run (NVT) followed by 100 ps in the microcanonical ensemble (NVE). The right panel zooms in on the numerical fluctuations of the total energy in the NVE run.

SUPPLEMENTARY FIGURE 7

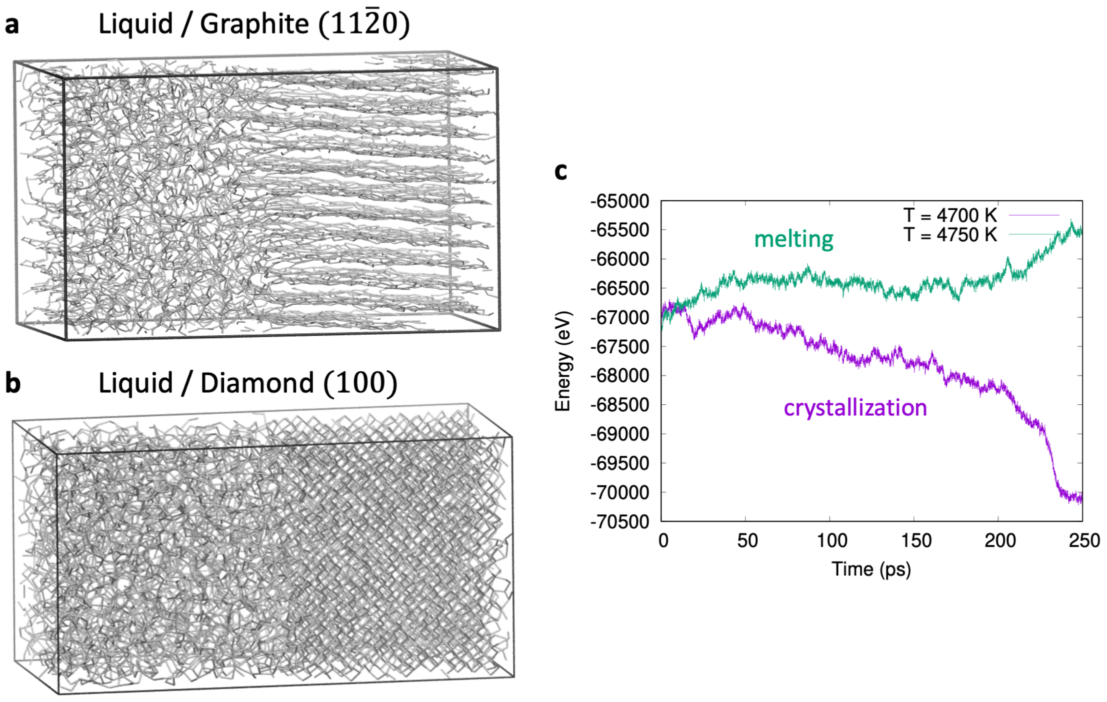

FIG. S7. (a, b) Two-phase liquid-solid simulation cells used to calculate the graphite and the diamond melting lines. The graphite-liquid simulation box was constructed by joining a supercell of graphite with a cell of liquid carbon equilibrated at 5000 K at the (100) plane. The same procedure was adopted for the diamond-liquid system. Both models contain $\sim 10,000$ atoms. (c) Potential energy during two-phase simulations of graphite crystallisation (T=4700 K) and melting (T=4750 K) at 10 GPa with the NEP@OptB88-vdW model. The melting point is between the two temperatures.

SUPPLEMENTARY FIGURE 8

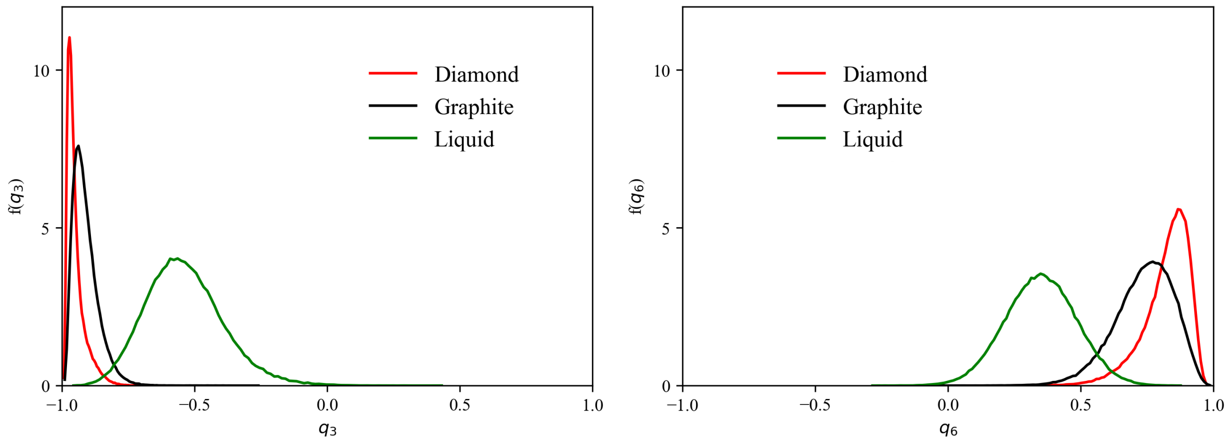

FIG. S8. Distribution of local bond-order parameter (a) q_3 and (b) q_6 for diamond, graphite, and liquid carbon at 4000 K and 15 GPa, computed according to the definitions given in Ref. 6.

SUPPLEMENTARY TABLE 1

TABLE I. Structural and elastic properties of graphite and diamond. The elastic constants of diamond were measured by resonant-ultrasound spectroscopy and those of graphite by inelastic X-ray scattering.[7,8]

Diamond	NEP	LDA[9]	Experiments[7]
a (Å)	3.54	3.55	3.61
ω_{LO} (THz)	39.1	x	39.9
C_{11} (GPa)	1116	1106	1079
C_{12} (GPa)	144	149	127
C_{44} (GPa)	594	593	578

Graphite	NEP	LDA[10]	Experiments[8]
a (Å)	2.44	2.44	2.46
c (Å)	6.60	6.63	6.71
ω_{LO} (THz)	42.8	47.7	47.4
C_{11} (GPa)	1128	1118	1109
C_{12} (GPa)	179	235	139
C_{33} (GPa)	36.0	29.5	38.7
C_{44} (GPa)	7.04	4.5	4.95

SUPPLEMENTARY TABLE 2

TABLE II. Hyperparameters for training the NEP@LDA and NEP@OptB88 models, all using full batch.

Hyperparameter	NEP-LDA-0 (Fan *et al.*[11])	NEP-LDA-1 (This work)	NEP-LDA-2 (This work)	NEP-LDA-3 (This work)	NEP-OptB88vdW (This work)
NEP version	3	3	3	3	4
r_c^R (Å)	4.2	4.2	4.2	5	8
r_c^A (Å)	3.7	3.7	3.7	4	4
n_{max}^R	10	10	12	12	12
n_{max}^A	8	8	8	8	8
N_{bas}^R	10	10	16	16	16
N_{bas}^A	8	8	12	12	12
l_{max}^{3body}	4	4	4	4	4
l_{max}^{4body}	2	2	2	2	2
l_{max}^{5body}	1	1	1	1	1
λ_1	0.05	0.05	0.05	0.05	0.0
λ_2	0.05	0.05	0.05	0.05	0.0850823
λ_e	1	1	1	1	1
λ_f	1	1	1	1	1
λ_v	0.1	0.1	0.1	0.1	0.1
N_{neu}	100	100	100	100	100
Population	50	50	50	50	100
Generation	250000	2000000	2000000	2000000	2000000

SUPPLEMENTARY NOTE 1

Hyperparameters for training the NEP@LDA model.

The relevant hyperparameters for the NEP@LDA model (NEP-LDA-0), as specified in the `nep.in` input file, are given below.

```
type        1 C
version     3
cutoff      4.2 3.7
n_max       10 8
basis_size  10 8
l_max       4 2 1
neuron      100
lambda_1    0.05
lambda_2    0.05
lambda_e    1.0
lambda_f    1.0
lambda_v    0.1
batch       100000
population  50
generation  250000
```

Hyperparameters for training the NEP@OptB88 (NEP-OptB88vdW) model.

The relevant hyperparameters for the NEP@OptB88 model, as specified in the `nep.in` input file, are given below.

```
type         1 C
version      4
cutoff       8  4
n_max        12  8
basis_size   16 12
l_max        4 2 1
neuron       100
lambda_1     0.0
```

```
lambda_e      1.0
lambda_v      0.1
batch         8000
population    100
generation    2000000
```

SUPPLEMENTARY REFERENCES

[1] V. L. Deringer and G. Csányi, Phys. Rev. B **95**, 094203 (2017).

[2] J. Klimeš, D. R. Bowler, and A. Michaelides, J. Phys.: Condens. Matter **22**, 022201 (2010).

[3] P. Rowe, V. L. Deringer, P. Gasparotto, G. Csányi, and A. Michaelides, J. Chem. Phys. **153**, 034702 (2020).

[4] A. Kondratyev and A. Rakhel, Phys. Rev. Lett. **122**, 175702 (2019).

[5] M. French and T. R. Mattsson, J. Appl. Phys. **116**, 013510 (2014).

[6] T. Li, D. Donadio, and G. Galli, J. Chem. Phys. **131**, 224519 (2009).

[7] A. Migliori, H. Ledbetter, R. G. Leisure, C. Pantea, and J. B. Betts, J. Appl. Phys. **104**, 053512 (2008).

[8] A. Bosak, M. Krisch, M. Mohr, J. Maultzsch, and C. Thomsen, Phys. Rev. B **75**, 153408 (2007).

[9] M. Barhoumi, D. Rocca, M. Said, and S. Lebègue, Solid State Commun. **324**, 114136 (2021).

[10] N. Mounet and N. Marzari, Phys. Rev. B **71**, 205214 (2005).

[11] Z. Fan, Y. Wang, P. Ying, K. Song, J. Wang, Y. Wang, Z. Zeng, K. Xu, E. Lindgren, J. M. Rahm, A. J. Gabourie, J. Liu, H. Dong, J. Wu, Y. Chen, Z. Zhong, J. Sun, P. Erhart, Y. Su, and T. Ala-Nissila, J. Chem. Phys. **157**, 114801 (2022).
